# Inhalation of welding fumes reduced sperm counts and high fat diet reduced testosterone levels; differential effects in Sprague Dawley and Brown Norway rats

**DOI:** 10.1186/s12989-019-0334-0

**Published:** 2020-01-10

**Authors:** Astrid Skovmand, Aaron Erdely, James M. Antonini, Timothy R. Nurkiewicz, Mohammad Shoeb, Tracy Eye, Vamsi Kodali, Katrin Loeschner, Janja Vidmar, Jørgen S. Agerholm, Sandra Goericke-Pesch, Ulla Vogel, Karin S. Hougaard

**Affiliations:** 1 The National Research Centre for the Working Environment, Lersø Parkallé, DK-2100 Copenhagen Ø, Denmark; 2 Department of Veterinary Clinical Sciences, University of Copenhagen, Dyrlægvej 16, DK-1870 Frederiksberg C, Denmark; 3 National Institute for Occupational Safety and Health, Morgantown, WV USA; 4 Department of Physiology and Pharmacology, West Virginia University, Morgantown, WV USA; 5 Center for Inhalation Toxicology, West Virginia University, Morgantown, WV USA; 6 National Food Institute, Technical University of Denmark, Kemitorvet 201, DK-2800 Kgs. Lyngby, Denmark; 7 Reproductive Unit of the Clinics – Clinic for Small Animals, University of Veterinary Medicine, Foundation, Bünteweg 15, 30559 Hannover, Germany; 8 Department of Health Technology, Technical University of Denmark, Ørsteds Pl., DK-2800 Kongens Lyngby, Denmark; 9 Department of Public Health, University of Copenhagen, Øster Farimagsgade 5, DK-1014 Copenhagen K, Denmark

## Abstract

**Background:**

Previous studies have shown that inhalation of welding fumes may induce pulmonary and systemic inflammation and organ accumulation of metal, to which spermatogenesis and endocrine function may be sensitive. Also obesity may induce low-grade systemic inflammation. This study aimed to investigate the effects on sperm production of inhaled metal nanoparticles from stainless steel welding, and the potential exacerbation by intake of a high fat diet. Both the inbred Brown Norway and the outbred Sprague Dawley rat strains were included to study the influence of strain on the detection of toxicity. Rats were fed regular or high fat (HF) diet for 24 weeks and were exposed to 20 mg/m^3^ of gas metal arc-stainless steel (GMA-SS) welding fumes or filtered air for 3 h/day, 4 days/week for 5 weeks, during weeks 7–12. Outcomes were assessed upon termination of exposure (week 12) and after recovery (week 24).

**Results:**

At week 12, the GMA-SS exposure induced pulmonary inflammation in both strains, without consistent changes in markers of systemic inflammation (CRP, MCP-1, IL-6 and TNFα). GMA-SS exposure lowered daily sperm production compared to air controls in Sprague Dawley rats, but only in GMA-SS Brown Norway rats also fed the HF diet. Overall, HF diet rats had lower serum testosterone levels compared to rats on regular diet. Metal content in the testes was assessed in a limited number of samples in Brown Norway rats, but no increase was obsedrved. At week 24, bronchoalveolar lavage cell counts had returned to background levels for GMA-SS exposed Sprague Dawley rats but remained elevated in Brown Norway rats. GMA-SS did not affect daily sperm production statistically significantly at this time point, but testicular weights were lowered in GMA-SS Sprague Dawley rats. Serum testosterone remained lowered in Sprague Dawley rats fed the HF diet.

**Conclusion:**

Exposure to GMA-SS welding fumes lowered sperm production in two strains of rats, whereas high fat diet lowered serum testosterone. The effect on sperm counts was likely not mediated by inflammation or lowered testosterone levels. The studied reproductive outcomes seemed more prone to disruption in the Sprague Dawley compared to the Brown Norway strain.

## Background

Inhalation of welding fumes is a serious occupational hazard for individuals working directly or in the vicinity of welding processes without proper personal protection. In 2017, the International Agency for Research on Cancer (IARC) classified welding fumes as a Group 1 carcinogen, i.e. ‘carcinogenic to humans’ [1]. Inhalation is the main route of exposure, making the airways an important target organ and site of absorption of the metal particles present in welding fumes. Respiratory illnesses, such as bronchitis, pulmonary fibrosis and “metal fume fever”, are common afflictions among welders [2]. Metal fume fever is characterized by influenza-like symptoms, influx of polymorphonuclear neutrophils granulocytes (PMNs) to the bronchoalveolar spaces, release of inflammatory mediators, and formation of reactive oxygen species [3, 4]. Long-term inhalation of welding fumes has been associated with low-grade lung and systemic inflammation and accumulation of metals in organs other than the lungs in humans; and may therefore potentially affect other organs than the port of entry [5–7].

Male welders have been reported to have reduced reproductive function in terms of poor sperm quality, decreased fecundity, changed levels of reproductive hormones, and increased risk for spontaneous abortion in their partners compared to unexposed individuals in some studies [8–12]. Other studies found little or no association between welding fume exposure and fertility [10, 13, 14]. This discourse may be due to the fact that poor fertility, including low sperm counts, are multifactorial. The interactions between genetics, lifestyle factors and occupational exposure to toxicants may be overlooked and hide true associations [15, 16]. Potential mechanisms underlying lung exposure to metal particles and reduced reproductive function in males have been proposed to involve a combination of direct toxicity of metals and particles and indirect toxicity due to lung inflammation arising from particles deposited in the lung and the subsequent release of pro-inflammatory mediators into the blood, as both metals and inflammation may interfere with testicular function [17]. Circulating metals and pro-inflammatory mediators may also interfere with the hypothalamic-pituitary-gonadal axis (HPG-axis) and disturb hormonal homeostasis and signaling to the testes, implying a potential for disturbance of spermatogenesis [18–20]. In experimental animals, a total of 28 weekly intratracheal instillations of Sprague Dawley rats with 2 mg of particles/instillation from either manual metal arc-hard surfacing (MMA-HS) or gas metal arc-mild steel (GMA-MS) significantly reduced sperm counts and serum prolactin levels. GMA-MS furthermore increased testosterone levels [21]. Increased levels of Zn, and Zn and Mn were found in the pituitary gland of the GMA-MS and MMA-HS exposed rats, respectively [21]. With fewer instillations of MMA-HS (once weekly for 7 weeks), increased levels of Cr and Mn were found in the testes [21]. Dosing by intratracheal instillation entails a high dose rate and may therefore induce higher lung inflammation compared to dosing by inhalation. High intake of fats and associated obesity has also been linked to male infertility. Potential underlying mechanisms include endocrine changes, for example decreased testosterone levels as reported previously [22–24]. A high-fat diet may also induce chronic low grade inflammation characterized by increased blood levels of cytokines such as IL-6 and TNFα, which may also interfere with testicular function [25, 26].

Male reproductive parameters as well as sensitivity to chemical exposures may vary with genotype and between in- and outbred strains [27–29]. Inbred strains of rodents are furthermore genetically more uniform compared to outbred strains. Hence, outbred strains may display larger phenotypic variation, which may lower statistical power and reproducibility [28, 30]. In selection of an animal model for testing for reproductive toxicity, it is therefore important that the species and strain are not resistant to the chemical exposure in question.

Here we have studied the hypotheses that a) inhalation of welding fume decreases testicular sperm counts and b) that high fat diet increases the effects of the welding fume inhalation on sperm counts by increasing systemic inflammation and/or decreasing testosterone levels. Both an inbred and an outbred strain of rat were exposed to welding fume and the diet high in fat to study whether the choice of animal strain could affect detection of effects on sperm count, if any. If welding fumes and high fat diet decreases sperm counts, different mechanisms may be at play. Hence welding fume inhalation and high fat diet may induce lung and systemic inflammation, and the solubilty of welding derived metal nanoparticles in welding fumes can result in the uptake and accumulation of metals in in the testes, all factors that may indirectly or directly interfere with sperm production.

## Methods

### Animals and experimental design

All animal procedures were reviewed and approved by the CDC-Morgantown Institutional Animal Care and Use Committee. The animal facilities were specific pathogen-free, environmentally controlled, and accredited by AAALAC, International. Male Sprague Dawley (Hla: (SD, BN) CVF; Hilltop Lab Animals, Scottdale, PA, USA) and Brown Norway rats (BN/RijHsd; Harlan Laboratories, Inc., Indianapolis, IN) and were free of viral pathogens, parasites, mycoplasmas, *Helicobacter* sp., and CAR Bacillus. All rats arrived at 5 weeks of age, but the Brown Norway rats were supplied in two different batches with some differences in body weights. The rats were acclimated for 5 days and were provided tap water, HEPA-filtered air, and irradiated Teklad 2918 standard diet composed of 18.6% protein, 44.2% carbohydrate, and 6.2% fat (Envigo Teklad Diets, Madison, WI, USA) ad libitum. After the acclimation 48 animals/strain continued on the standard Teklad 2918 diet and 48 animals/strain were fed a Teklad Custom 44.6% Fat Kcal Western Diet (Envigo Teklad Diets, Madison WI, USA) ad libitum. The HF Western diet was composed of 14.8% protein, 40.6% carbohydrate, and 44.6% fat; and is designed to mimic the western diet with a content of 21% anhydrous milk fat and 34% sucrose. Soybean (2%) was included to supplement essential fatty acids.

**Fig. 1:**
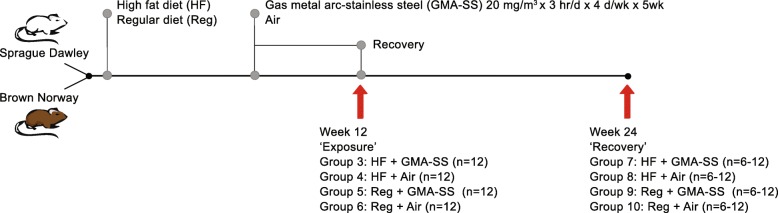
After acclimation, the Sprague Dawley and Brown Norway rats were fed either a high fat or a regular diet. At week 7, the exposure to either GMA-SS welding fumes (20 mg/m^3^ × 3 h/d × 4 d/wk. × 5 wk) or HEPA filtered air began. At week 12, GMA-SS exposure were terminated and half of the rats were humanely sacrificed (*n* = 12/group/strain). For the other half the recovery period continued for another 12 weeks, before the animals were sacrificed and organs sampled at week 24 (*n* = 6/group for Sprague Dawley; *n* = 12/group for Brown Norway). Red arrows indicate sampling of groups

The Sprague Dawley and Brown Norway rats continued on their respective diets for 7 weeks, where the two diet groups were further divided into 4 subgroups. One HF diet group and one regular diet group were exposed to a target concentration of 20 mg/m^3^ of gas metal arc-stainless steel (GMA-SS) welding fumes for 3 h a day, 4 days a week, for 5 weeks. The remaining two groups served as exposure controls and underwent the same exposure but with HEPA filtered air. At week 12, following the 5 week exposure period, a set of rats from each exposed group (*n* = 12) were humanely sacrificed by sodium pentobarbital overdose via intraperitoneal injection (> 100 mg/kg body weight; Fatal-Plus Solution, Vortech Pharmaceutical, Inc., Dearborn, MI) followed by exsanguination of the abdominal aorta. The remaining rats were allowed to recover for 12 weeks until week 24 when they were humanly sacrificed as described above (*n* = 12; Brown Norway rats). Body weights were recorded at baseline and at 4, 12 and 24 weeks of the study. Samples were stored at − 80 °C until analysis. Due to unforeseen reasons, only half of the samples for the Sprague Dawley rats were collected at week 24 (*n* = 6). The timeline of the study and the groups are presented in Fig. 1.

### Welding fume generator system and exposure chamber

The welding fume generator system and exposure chamber has been previously described [31]. The welding chamber was divided into three compartments: an enclosed control room, an robotic welding fume generator compartment and the animal whole body exposure chamber, also holding the fume and gas characterization equipment. The welding fume generation system consisted of a welding power source (Power Wave 455, Lincoln Electric, Cleveland, OH, USA), an automated and programmable six-axis robotic arm (Model 100 Bi, Lincoln Electric, Cleveland, OH, USA), a water-cooled arc welding torch (WC 650 A, Lincoln Electric, Cleveland, OH, USA), a wire feeder that supplied the wire to the torch at a programmed rate, and an automatic welding torch cleaner to keep the welding nozzle free of debris. The gas metal arc welding was performed using a stainless steel electrode (Blue Max E308LSi wire, Lincoln Electric, Cleveland, OH, USA). Welding took place on A36 carbon steel plates for daily exposures of 3 h at 25 V and 200 A. During welding, a shielding gas combination of 95% Ar and 5% CO_2_ (Airgas Co., Morgantown, WV, USA) was continually delivered to the welding nozzle. A flexible trunk was positioned approximately 45 cm from the arc to collect the generated fume and transport it to the exposure chamber. The generated welding fume was mixed with dry HEPA-filtered air. Continuous records of exposure chamber fume concentration, temperature, and humidity were maintained during exposure. Fume was collected onto 37-mm Teflon filters at a rate of 1 L/min, and the particle mass delivered to the exposure chamber was determined gravimetrically every 30 min in duplicate during the daily 3 h exposure. The metal composition of the particles generated by the stainless steel welding system was assessed as described [31, 32], see Table 1 for results.

**Table 1:** Metal composition of stainless steel welding fumes

Metal	Weight % metal
Iron	52.7% ± 0.3
Chromium	16.7% ± 0.3
Manganese	24.3% ± 0.5
Nickel	5.8% ± 0.0
Copper	0.4% ± 0.0

Mean weight % ± SD (*n* = 3)

### Bronchoalveolar lavage fluid

Bronchoalveolar lavage fluid (BALF) was collected as previously described [33]. In brief, the main bronchus of the right lung was lavaged with 6 ml aliquots of PBS until 30 ml were collected. The samples were centrifuged for 10 min at 500 g, and the cell-free BALF discarded. The cell pellets from all washes for each rat were combined, washed, and resuspended in 1 ml of PBS buffer. The total cell number were determined using Coulter Multisizer II and AccuComp software (Coulter Electronics, Hialeah, FL, USA). The cells were differentiated using a Cytospin 3 centrifuge (Shandon Life Sciences International, Cheshire, UK). Cell suspensions were spun for 5 min at 800 rpm and pelleted onto a slide. Cells (200/rat) were identified after labeling with Leukostat stain (Fisher Scientific, Pittsburgh, PA, USA) as alveolar macrophages (AMs) and PMNs.

### Serum levels of inflammatory markers

Whole blood was obtained at sacrifice for serum collection at 12 and 24 weeks. In Sprague-Dawley rats. Levels of C-reactive protein (CRP), monocyte chemoattractant protein 1 (MCP-1), interleukin 6 (IL-6) and tumor necrosis factor alpha (TNFα) in serum were quantified using enzyme-linked immunosorbent assay (ELISA) as per the manufacturer’s recommendation (Invitrogen, catalogue 88,750,128, KRC 1012, KRC 0062 and KRC 3012).

### Sperm production

Initially the adipose tissue around the right frozen testes was trimmed and the tunica albuginea was removed by making a shallow longitudinal incision before peeling it away with forceps. The testes were weighed and placed into 30 ml of 0.05% TRITON-X100 and homogenized for 5 min using the IKAULTRA TURRAX T25 disperser S25 N-10G. Homogenates were kept on ice for 30 min. Two hundred microliters of the homogenate were mixed with 200 μl of 0.04% trypan blue and left for 5 min at room temperature before loading onto an improved Neubauer chamber [34]. Counts of sperm heads were done in triplicates and averaged. The daily sperm production (DSP) was calculated using the formula DSP = N / 6.10, where N is the total number of spermatids per sample calculated using a standard counting chamber formula and 6.10 represents the number of days for a spermatid to develop through stages 14 to 16 in rats, i.e. the stages where spermatids are resistant to homogenization [35]. Sperm content per gram of testes (SC/G_testes_) was calculated by dividing total sperm counts with the testicular weight. Two testes samples from the Brown Norway rats were discarded from the study because they were void of sperm.

### Testosterone

The serum testosterone level was determined in duplicates using a competitive ELISA according to the manufacturer’s protocol (RTC001R, Biovendor, Brno, Czech Republic). The standard curve was in the range of 0.1–25 ng/ml.

### Analysis of metal content

The left testes (1–2 g wet mass) of each rat was weighed in quartz tubes, and 4 ml of concentrated nitric acid (PlasmaPure 67–69% HNO_3_, SCP Science, USA) was added. The samples were left overnight in fume hood for pre-digestion. The next day, the samples were digested using the microwave reaction system Multiwave 3000 (Anton Paar GmbH, Austria) at elevated temperature and pressure, using the following program: ramp to temperature = 220 °C for 25 min, hold at temperature = 220 °C, max. 80 bar for 15 min, cool down for 20 min. After digestion, the samples were diluted to a final mass of 20 g with ultrapure water (18.2 MΩ·cm at 25 °C and max. 5 ppb total organic carbon, Merck Milli-Q Integral 5, USA). For quality assurance, blanks and reference material (SRM 1577c Bovine liver, NIST, MA, USA) were included in the analysis. For digestion of the reference material, 0.200 ± 0.005 g of the sample (dry mass) were used. Prior to ICP-MS analysis, the digested samples were diluted 2.5 times with ultrapure water. Determination of the mass concentrations for the selected elements was performed based on external calibration by measuring multi-element standards in the concentration range of 0.5–1000 μg/l with online internal standardization (25 μg/l solution of Sc, Y and Rh). Calibration standards were prepared from a multi-element stock solution with 10 mg/l elemental concentration, while internal standards were made from individual standard solutions that contained 1000 mg/l of each element (standards provided by Plasma CAL, SCP Science). All standards were matrix-matched with the diluted samples (i.e. prepared in 5.4% HNO_3_). For ICP-MS analysis, an Agilent 8900 ICP-QQQ-MS (Agilent Technologies, CA, USA) equipped with a Micro Mist borosilicate glass concentric nebulizer, a Scott type double-pass water-cooled spray chamber, platinum cones and an auto sampler (SPS4, Agilent Technologies) was used. The analysis was performed in single quadrupole mode with helium as a cell gas (5 ml/min for isotopes ^55^Mn, ^56^Fe, ^60^Ni and ^63^Cu) and in mass-shift mode with oxygen as cell gas (approximately 0.45 ml/min for isotope ^52^Cr ➔ ^52^Cr^16^O).

### Statistics

Two-way analysis of variance (ANOVA) was conducted to investigate the effects of GMA-SS exposure, diet type and the interaction between the two on body weight gain, BALF cell counts, inflammatory markers, testicular weights, DSP, SC/Gtestes, serum testosterone and metal content in the testes. Prior to analysis, the data distribution was tested with a Shapiro-Wilk test, data that was not normally distributed was log-transformed for analysis. Provided the overall ANOVA indicated significant difference, groups were compared by Fisher’s Least Significant Difference test. Samples that were three deviations away from the mean were removed from the analysis. The statistical analysis was performed using Origin Pro, version 2017 (64-bit), OriginLab Corp (Northampton, MA, USA).

## Results

**Fig. 2:**
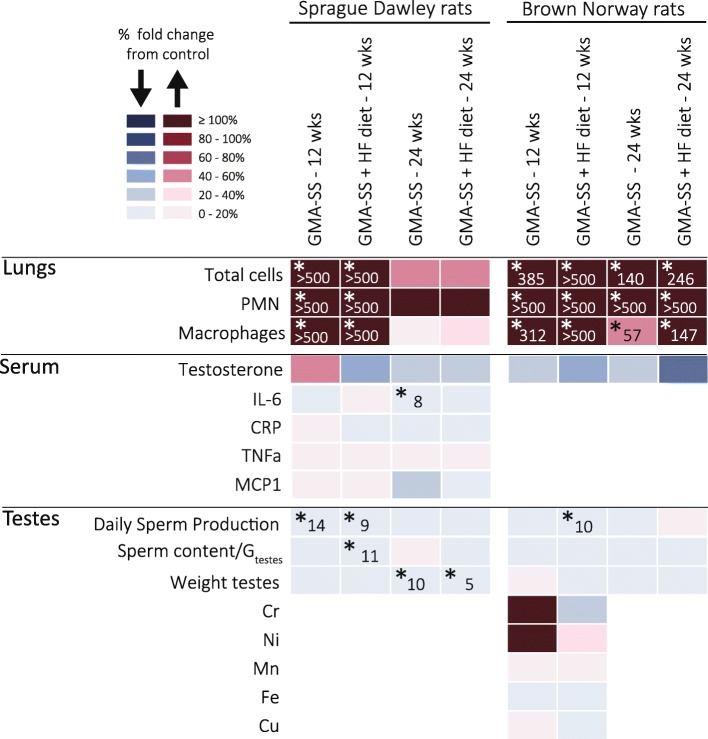
Summary of results for Sprague Dawley and Brown Norway rats fed regular or HF diet, at week 12 and 24. Color change indicates % change from Air+Reg diet calculated as (B-A)/A*100 (A = control & B = exposure). Blue color indicates decrease and red indicates increase from Air+Reg diet. Statistically significant results are indicated by the level of significance and % change from Air+Reg diet (*p < 0.05)

Figure 2 provides an overview of the results. *P*-values from the two-way ANOVAs are given in Additional file 2: Tables S1-S4.

### Bronchoalveolar lavage fluid cell counts

BALF cell counts were performed to determine pulmonary inflammation in terms of neutrophil influx. The overall outcome of the two-way ANOVA are shown in Additional file 2: Table S1.

#### Sprague Dawley: 12 weeks – exposure

The overall two-way ANOVA showed a statistically significant effect of GMA-SS for total cell counts, AMs and PMNs in BALF, and for PMNs, furthermore statistical significance of diet and the interaction between GMA-SS and diet. In accordance, total cell counts, AMs and PMNs in BALF were significantly elevated in the GMA-SS exposed rats compared to the air controls (*p* < 0.05). Interestingly, the GMA-SS + Reg diet rats had a statistically significantly higher number of PMNs in BALF compared to the GMA-SS + HF diet group, indicating that the diet high in fats modulated the lung inflammatory response in a downward direction (Table 2).

**Table 2:** Bronchoalveolar lavage fluid cell counts in Sprague Dawley rats

	Total cell number (10^6^)	Macrophages (10^6^)	Neutrophils (10^6^)
Week 12 – exposure			
Air + Reg	3.83 + 0.4	3.83 + 0.4	0.00 + 0.0
Air + HF	4.46 + 0.5	4.46 + 0.5	0.00 + 0.0
GMA-SS + Reg	**92.1** **+** **13.0** ^**ab**^	**77.6** **+** **11.0** ^**ab**^	**14.5** **+** **5.2** ^**abc**^
GMA-SS + HF	**68.0** **+** **9.7** ^**ab**^	**65.3** **+** **9.7** ^**ab**^	**2.71** **+** **1.3** ^**ab**^
Week 24 – recovery			
Air + Reg	4.66 + 0.5	4.65 + 0.5	0.01 + 0.01
Air + HF	3.51 + 0.4	3.50 + 0.4	0.01 + 0.01
GMA-SS + Reg	**6.94** **+** **1.1**^**b**^	4.99 + 0.5	0.16 + 0.01
GMA-SS + HF	4.92 + 0.8	4.87 + 0.7	0.06 + 0.02

Total cell number, macrophages and neutrophils in the bronchoalveolar lavage fluid from Sprague Dawley rat lungs at week 12 after a 5-week exposure to 20 mg/m^3^ of GMA-SS; and at week 24 after a 12 week recovery period. Mean ± SD (*n* = 6). Significant effects are highlighted in bold

^a^Significantly different from Air+Reg diet (*p* < 0.05)

^b^Significantly different from Air+HF diet (*p* < 0.05)

^c^Significantly different from GMA-SS + HF diet (*p* < 0.05)

#### Sprague Dawley: 24 weeks – recovery

At this time point the overall ANOVA showed significant effects of only the total cell counts for both exposures, as AM and PMN counts in BALF in the GMA-SS exposed rats had returned to air control levels. Pair wise comparisons showed that total cell counts in BALF in the GMA-SS + Reg diet rats remained slightly and significantly elevated compared to the Air+HF diet rats group, supporting the modulation of lung inflammation by the HF diet (*p* < 0.05, Table 2).

#### Brown Norway: 12 weeks – exposure

The overall two-way ANOVA showed a statistically significant effect of GMA-SS and diet for total cell counts, AMs and PMNs in BALF, and for PMNs furthermore statistically significant interaction between GMA-SS and diet. On a pair wise basis, total cell counts, AMs and PMNs in BALF were significantly elevated in the GMA-SS exposed rats compared to both air control groups (*p* < 0.05). In contrast to the Sprague Dawley rats, there was no significant difference between the numbers of PMNs in BALF in the GMA-SS + Reg diet compared to the GMA-SS + HF diet group (Table 3).

**Table 3:** Bronchoalveolar lavage fluid cell counts in Brown Norway rats

	Total cell number (10^6^)	Macrophages (10^6^)	Neutrophils (10^6^)
Week 12 – exposure			
Air + Reg	12.8 + 1.2	12.7 + 1.1	0.14 + 0.1
Air + HF	8.7 + 0.2	8.7 + 0.2	0.00 + 0.0
GMA-SS + Reg	**62.1** **+** **4.4** ^**ab**^	**52.4** **+** **4.2** ^**ab**^	**9.66** **+** **2.6** ^**ab**^
GMA-SS + HF	**76.5** **+** **4.7** ^**ab**^	**68.9** **+** **3.7** ^**ab**^	**7.39** **+** **1.3** ^**ab**^
Week 24 – recovery			
Air + Reg	18.0 + 2.3	18.0 + 2.3	0.04 + 0.04
Air + HF	14.4 + 3.3	14.4 + 3.3	0.00 + 0.0
GMA-SS + Reg	**43.2** **+** **3.1** ^**ab**^	**28.4** **+** **2.5** ^**ab**^	**14.9** **+** **2.1** ^**ab**^
GMA-SS + HF	**49.9** **+** **6.6** ^**ab**^	**35.7** **+** **4.1** ^**ab**^	**14.2** **+** **2.8** ^**ab**^

Total cells number, macrophages and neutrophils in the bronchoalveolar lavage fluid from Brown Norway rat lungs at week 12 after a 5-week exposure to 20 mg/m^3^ of GMA-SS, and at week 24 after a 12 week recovery period. Mean ± SD (*n* = 6). Significant effects are highlighted in bold

^a^Significantly different from Air+Reg diet (*p* < 0.05)

^b^Significantly different from Air+HF diet (*p* < 0.05)

#### Brown Norway: 24 weeks – recovery

Two-way ANOVA showed significant effect of GMA-SS exposure for all. Total cell and AM counts in BALF for the GMA-SS exposed rats had decreased during the recovery period, but remained statistically significantly elevated as compared to both air control groups. In contrast, the influx of PMNs appeared to have continued during the 12 weeks after the last exposure, such that the numbers of PMNs in BALF were almost doubled in the GMA-SS groups as compared to immediately after end of exposure and remained significantly elevated compared to air controls (Table 3).

### Serum levels of inflammatory markers

Serum levels of inflammatory markers (IL-6, CRP, MCP-1 and TNFα) were assessed in Sprague-Dawley rats at 12 and 24 weeks (for *p*-values for the overall ANOVA, see Additional file 2: Table S2). The overall analyses did not indicate significant effects of GMA-SS nor HF diet relative to TNFα.

#### Sprague Dawley: 12 weeks – exposure

The only maker for which the overall ANOVA indicated significant effects at 12 weeks were IL-6, probably due to the numerically, slightly higher IL-6 serum levels in the HF groups compared to the groups on regular diet, but only the IL-6 levels in the Air+HF group were statistically significantly higher compared to both of the regular diet groups (0.01 < *p* < 0.05).

#### Sprague Dawley: 24 weeks – recovery

**Fig. 3:**
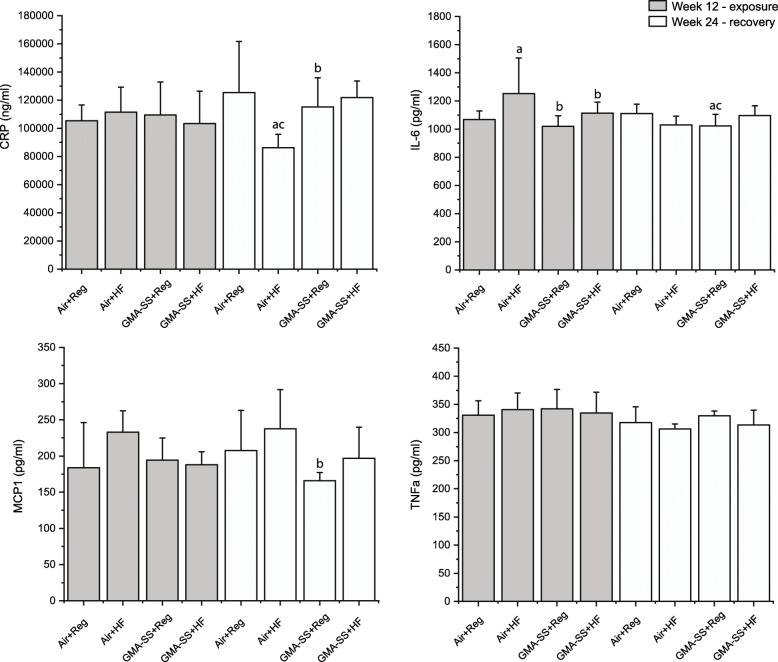
C-reactive protein (CRP), interleukin 6 (IL-6), monocyte chemoattractant protein 1 (MCP-1), and tumor necrosis factor alpha (TNFα) in serum of Sprague Dawley rats at week 12 following 5 weeks of exposure to 20 mg/m^3^ of GMA-SS, and at week 24 following 12 weeks of recovery. Mean ± standard error (*n* = 6). ^a^ Significantly different from Air+Reg diet (*p* < 0.05); ^b^ Significantly different from Air+HF diet (*p* < 0.05); ^c^ Significantly different from GMA-SS + HF diet (*p* < 0.05)

For IL-6, the interaction of GMA-SS with diet was statistically significant, and pairwise comparisons showed that the GMA-SS + Reg diet group had lower serum levels of IL-6 than the Air controls (*p* < 0.05, Fig. 3). For CRP, the interaction between GMA-SS and diet was statistically significant. Pairwise comparisons showed that the level in the Air+HF diet group was significantly lowered compared to the three other groups (0.01 < *p* < 0.05, Fig. 3). For MCP-1 (Fig. 3), the overall ANOVA showed a statistically significant effect of GMA-SS at 24 weeks. Pairwise comparisons showed that the MCP-1 levels in the GMA-SS + Reg diet group was significantly lower than in the Air+HF diet group. In summary, we did not see any consistent effects of either exposure on the assessed markers of systemic inflammation.

### Testes weight, DSP and testosterone level

Testes weight, DSP, sperm content per gram of testes (SC/G_testes_) and serum testosterone levels were measured as reproductive endpoints. The overall outcome of the two-way ANOVA are shown in Additional file 2: Table S3.

#### Sprague Dawley: 12 weeks – exposure

**Fig. 4:**
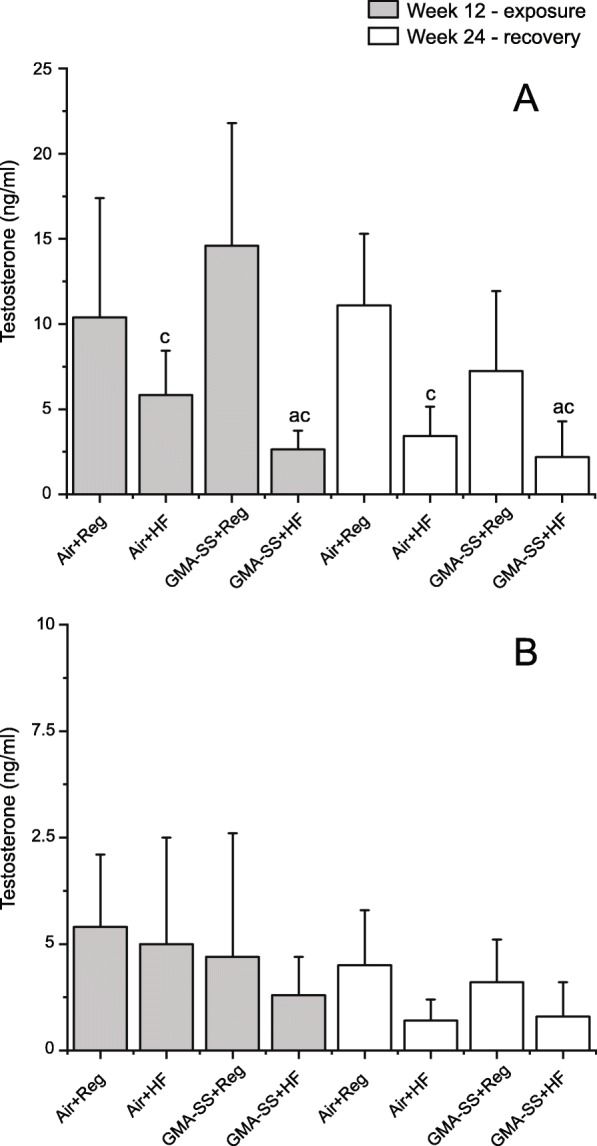
Testosterone (ng/ml) in serum of Sprague Dawley (**a**) and Brown Norway (**b**) rats at week 12 following weeks of exposure to 20 mg/m^3^ of GMA-SS, and at week 24 following 12 weeks of recovery. Mean ± standard error (*n* = 6–12). ^a^ Significantly different from Air+Reg diet (*p* < 0.05); ^c^ Significantly different from GMA-SS + Reg diet (*p* < 0.05)

The overall statistical analysis did not indicate differences in absolute testes weight. For DSP, the two-way ANOVA showed significant effect of GMA-SS. GMA-SS + Reg diet and GMA-SS + HF diet rats had significantly lower DSP compared to Air+Reg diet controls (*p* < 0.05, Table 4). GMA-SS + Reg diet rats had also significantly lower DSP compared to Air+HF diet males (*p* < 0.05, Table 4). For sperm content per gram of testes (SC/G_testes_), the overall ANOVA indicated statistically significant effects of GMA-SS exposure. Levels were similar in the two GMA-SS groups, but only the GMA-SS + Reg diet rats had statistically significantly lower SC/G_testes_ compared to the Air+Reg and Air+HF groups (*p* < 0.05, Table 4). For serum testosterone, the overall ANOVA showed significant interaction of GMA-SS and diet. Pair wise comparisons showed that testosterone concentrations in the HF diet rats were significantly lower compared to regular diet fed rats, whereas no significant modulation of GMA-SS exposure was seen (*p* < 0.05, Fig. 4).

**Table 4:** Absolute testes weight, daily sperm production (DSP) and serum testosterone concentration in Sprague Dawley rats

	Absolute testes weight (g)	DSP (10^7^)	SC/G_testes_ (× 10^7^)
Week 12 – exposure			
Air + Reg	2.10 ± 0.2	1.39 ± 0.1	8.95 ± 1.2
Air + HF	2.02 ± 0.2	1.36 ± 0.1	9.09 ± 0.9
GMA-SS + Reg	2.06 ± 0.1	**1.19 ± 0.2** ^**ab**^	**7.90 ± 0.7**^**ab**^
GMA-SS + HF	1.98 ± 0.2	**1.23 ± 0.1** ^**a**^	7.75 ± 0.8
Week 24 – recovery			
Air + Reg	2.29 ± 0.1	1.31 ± 0.7	3.50 ± 0.2
Air + HF	2.15 ± 0.2	1.24 ± 0.2	3.51 ± 0.6
GMA-SS + Reg	**2.04 ± 0.2** ^**a**^	1.18 ± 0.1	3.53 ± 0.1
GMA-SS + HF	**2.03 ± 0.1** ^**a**^	1.14 ± 0.7	3.42 ± 0.2

Absolute testes weight (g), daily sperm production (10^7^) and sperm content per gram of testes in Sprague Dawley rats at week 12 after a 5-week exposure to 20 mg/m^3^ of GMA-SS, and at week 24 after a 12 week recovery period. Mean ± SD (*n* = 6–12). Significant effects are highlighted in bold

^a^Significantly different from Air+Reg diet (*p* < 0.05)

^b^Significantly different from Air+HF diet (*p* < 0.05)

#### Sprague Dawley: 24 weeks – recovery

The overall statistical analysis showed a near-significant effect of GMA-SS exposure on DSP, significant effects of GMA-SS exposure on testes weight and interaction of the two factors for testosterone levels. DSP remained low in both GMA-SS exposed groups compared to the air controls, however, the effect was not statistically significant. Testes weights of air controls had increased slightly in the air control groups at week 24, but not in GMA-SS exposed rats, and GMA-SS exposed rats now presented with significantly lower testes weights compared to the Air+Reg diet group (*p* < 0.05, Table 4). Serum testosterone of the GMA-SS + Reg diet rats decreased numerically from week 12 to week 24, and the HF diet fed rats continued to show significantly lower serum testosterone concentrations compared to the rats on regular diet (*p* < 0.05, Fig. 4) (Table 5).

**Table 5:** Absolute testes weight, daily sperm production (DSP) and serum testosterone concentration in Brown Norway rats

	Absolute testes weight (g)	DSP (10^7^)	SC/G_testes_ (×10^7^)
Week 12 – exposure			
Air + Reg	1.61 ± 0.1	2.70 ± 0.2	10.10 ± 0.7
Air + HF	1.57 ± 0.1	2.69 ± 0.2	10.30 ± 1.0
GMA-SS + Reg	1.64 ± 0.1	2.68 ± 0.1	9.94 ± 0.5
GMA-SS + HF	1.53 ± 0.1	**2.41 ± 0.1**^**abc**^	9.73 ± 0.8
Week 24 – recovery			
Air + Reg	1.65 ± 0.1	2.67 ± 0.2	10.2 ± 0.8
Air + HF	1.62 ± 0.1	2.62 ± 0.4	9.9 ± 1.7
GMA-SS + Reg	1.57 ± 0.1	2.56 ± 0.2	9.1 ± 3.0
GMA-SS + HF	1.60 ± 0.1	2.67 ± 0.3	9.9 ± 1.4

Absolute testes weight (g), daily sperm production, and sperm content per gram of testes in Brown Norway rats at week 12 following a 5-week exposure to 20 mg/m^3^ of GMA-SS, and at week 24 following a 12 week recovery period. Mean ± standard error (*n* = 6–12). Significant effects are highlighted in bold

^a^Significantly different from Air+Reg diet (*p* < 0.05)

^b^Significantly different from Air+HF diet (*p* < 0.05)

^c^Significantly different from GMA-SS + Reg diet (*p* < 0.05)

### Body weights

**Fig. 5:**
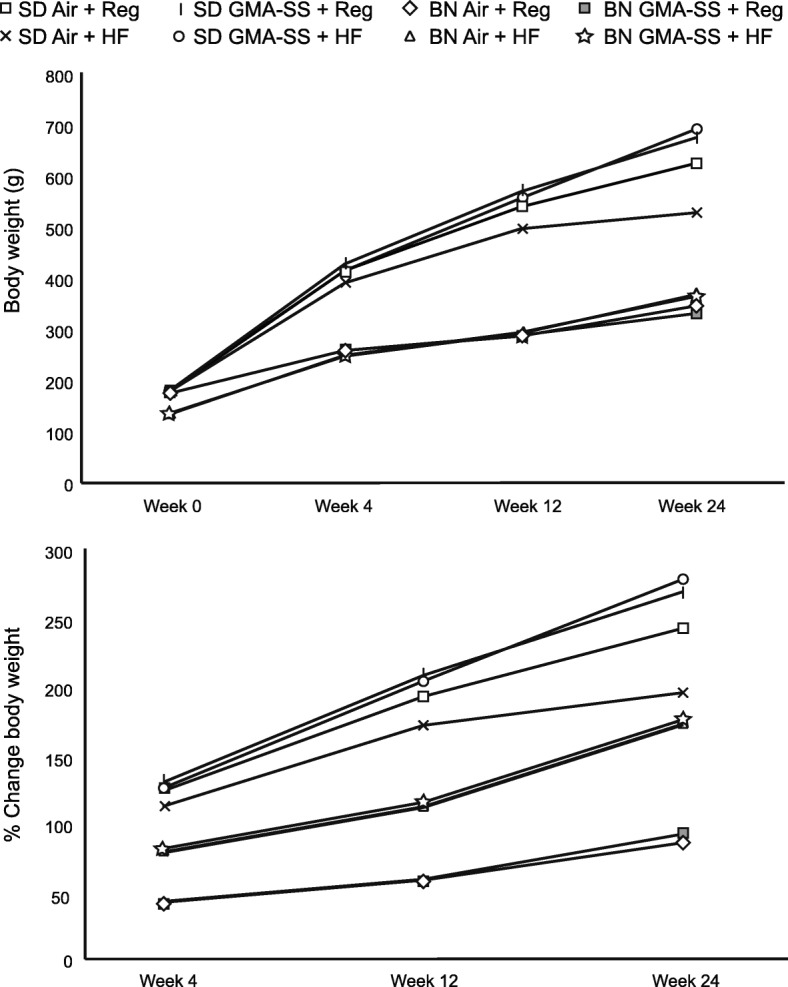
Body weight and relative change in body weight (%) comparing HF and regular diets for the Sprague Dawley and Brown Norway strains during the 24-wk study period. Starting wk. 7 during diet maintenance, groups of rats from each strain were exposed to GMA-SS welding fume (20 mg/m^3^ × 3 h/d × 4 d/wk. × 5 wk) or filtered air (control) until wk. 12 at which time animals from each strain were euthanized. A separate set of rats from each strain were allowed to recover from welding fume exposure until the end of the 24-wk period. Values are means + standard error (*n* = 12–24/group). See text for *p* values

For both rat strains, the HF diet affected body weight from baseline to 12 and 24 weeks of age (Fig. 5). The statistical analysis below has been performed for relative body weight gain between baseline and 12 weeks and between 12 and 24 weeks, but the outcomes were almost similar for actual body weights. The overall outcome of the two-way ANOVA are shown in Additional file 2: Table S4.

#### Sprague Dawley: 12 weeks – exposure

The statistical analysis showed highly significant effects of both GMA-SS exposure and HF diet on relative body weight gain compared to base line, as well as significant interaction between the two. The GMA-SS + Reg diet group gained statistically significantly relatively less weight than all of the three other groups (*p* < 0.001). There was no difference between relative weight gain in the two HF diet groups, nor between the GMA-SS + HF and Air+Reg diet groups, but the Air+Reg diet group gained relatively less than the Air+HF diet group.

#### Sprague Dawley: 24 weeks – exposure

The effects of HF diet and GMA-SS exposure on relative body weight gain compared to at 12 weeks were both statistically significant as was the interaction between the two (0.000 < *p* < 0.05). Apart from the two HF-diet groups, all groups now differed statistically significantly from each other. Overall, the HF diet increased body weight gain, but GMA-SS decreased body weight significantly only in the regular diet group.

#### Brown Norway: 12 weeks – exposure

At this time point, the HF diet increased the relative body weight gain compared to base line statistically significantly (*p* < 0.000).

#### Brown Norway: 24 weeks – recovery

HF diet had a highly significant effect on relative body weight gain compared to base line. Weight gain was similar in the two HF diet groups and in the two regular diet groups, but weight gain differed significantly between HF and regular diet groups.

### Metal content in testes of Brown Norway rats

Metal content was determined in the testes of Brown Norway rats (*n* = 5–6/group) at the end of exposure at week 12. There were no significant differences in the levels of Cr, Ni, Mn, Fe and Cu between groups (Additional file 1: Figure S1). The estimated limit of detection in the tissue samples was 2.5 μg/kg for Cr, 3.1 μg/kg for Mn, 28.3 μg/kg for Fe, 2.5 μg/kg for Ni and 44.2 μg/kg for Cu.

## Discussion

The present study investigated if inhalation of welding fume decreased testicular sperm counts, and furthermore whether HFD increased the effects of the welding fume inhalation. Figure 2 provides an overview of the results. There are approximately 80 different types of welding processes available for commercial use. They differ with respect to electrode coating, shielding gases, filler and base metals, paint and surface coating. Hence, each welding process generates particles with a unique combination of metals. In addition, working conditions, ventilation and welding skills can modify the composition and the generation rate of welding fumes [2]. Gas metal arc-stainless steel welding particles generated by the welding system were mainly composed of Fe, contained significant amounts of Cr and Mn and to a lesser extent Ni and Cu (Table 1). The particle size distribution have been previously characterized [33, 36] and particles distributed between100 nm to 1 μm in size with a mass median aerodynamic diameter calculated to 250 nm.

As expected, inhalation exposure to 20 mg/m^3^ of GMA-SS welding fumes for 3 h a day, 4 days a week for 5 weeks induced pulmonary inflammation in both the Sprague Dawley and Brown Norway strains. The GMA-SS exposure induced pulmonary inflammation in the Brown Norway rats that persisted throughout recovery. In the Sprague Dawley rats, GMA-SS also induced overt pulmonary inflammation at 12 weeks that subsided during recovery. There was interaction between diet and welding fume exposure in relation to neutrophil influx. Hence, the HF diet appeared to suppress the pulmonary inflammatory response induced by inhalation of the GMA-SS generated particles. C57BL/6 J mice intratracheally instilled with carbon black particles and also fed a HF diet mounted a lower level of macrophage inflammatory protein 2 response in BAL-fluid compared to particle-exposed mice on a low fat diet. This is indicative of suppression of macrophage pro-inflammatory activation, which could potentially decrease PMN influx in the HF diet fed rodents [37], but recruitment of PMNs upon carbon black instillation was however unaffected by the HF diet [37, 38].

The cellular inflammatory response in the lung following airway exposure to nanosized particles is often characterized by PMN influx to the bronchoalveolar space which correlates with induction of inflammatory cytokines [39]. Inflammatory mediators released to the blood may be transported to extra pulmonary organs and have been hypothesized to exert adverse effects on the testes and interfere with spermatogenesis in an indirect way [17, 35]. Here, we measured serum levels of TNFa, IL-6, CRP, and CCL2/MCP-1 in the Sprague Dawley rats. GMA-SS exposure did not increase serum levels of any of the cytokines, neither statistically significantly nor numerically. Thus, we saw no GMA-SS induced systemic inflammation.

The Sprague Dawley rats exposed to GMA-SS had significantly reduced DSP at week 12, at which time point there was a large cellular inflammatory response in the lungs. During the recovery period this inflammation subsided. DSP remained numerically lowered in the GMA-SS compared to air control groups, albeit not significantly so. It should, however, be kept in mind that the group size was 12 at week 12 but only 6 at week 24. At week 24, testicle weights were significantly lower in the GMA-SS groups compared to the air control groups. This may be indicative of degenerative changes in the testes [40], but it could also be speculated that populations of developing sperm cells (other than the homogenization resistant stages) could have been depleted or reduced and not yet recovered [41]. In the Brown Norway rats, large pulmonary inflammatory response was observed at week 12 and appeared to have increased at week 24, rather than subsided. DSP was only reduced in the GMA-SS group on HF diet in week 12, and testes weights were unaffected at both time points. In the Sprague Dawley rats, the decreased sperm production occurred concomitantly with massive cellular lung inflammation and is in principle compatible with an indirect effect of GMA-SS induced inflammation on spermatogenesis. We did, however, not observe any indications of systemic inflammation based on blood measurements in support hereof. In the Brown Norway rats, lung inflammation was persistent and even increasing throughout the study period without persistent depression of spermatogenesis. In fact, reduced DSP was only observed in the GMA-SS + HF diet group, i.e. the group with the lowest level of PMNs in lung fluid. The Brown Norway findings do therefore not support particle-induced airway inflammation as a major risk factor for male reproductive function, albeit it should be recognized that markers of systemic inflammation was not assessed in this strain. These findings indicate that the reduced sperm counts were not mediated by systemic inflammation resulting from pulmonary inflammation and that HF diet did not exacerbate the effects of GMA-SS exposure.

We have previously demonstrated that testes function assessed by measurements of several biomarkers of spermatogenesis did not change in NMRI mice exposed repeatedly by intratracheal instillations to 0.1 mg of two different carbon blacks (Printex 90 and Flammruss 101), graphene oxide, and diesel exhaust particles (SRM 1650b), in spite of overt and surface area-dependent PMN influx into the bronchoalveolar space [35]. Similar findings were recently reported for titanium dioxide nanoparticles and quartz particles [42]. Altogether, these findings suggest that pulmonary inflammation resulting from airway particle exposure does not affect sperm production per se. The present study is limited by the fact that for logistic reasons, testicular sperm counts of the homogenized testes and testicular weights are the only available endpoints for male reproduction. Future studies would benefit from inclusion of a wider range of outcomes for male reproductive function as well as for systemic inflammation.

In humans, several sperm parameters, including sperm concentration, have been shown to correlate negatively with blood content of metals like Cr among welders in India [43]. Chromium exposure has in several experimental animal studies resulted in increased ratios of abnormal spermatozoa and decreased sperm counts. Recent in vitro findings indicate that hexavalent Cr may impact differentiation and self-renewal of spermatogonial stem cells [44]. In rats, the metals generated by the GMA-SS welding system used here have been shown to clear from the lungs at different rates, which is indicative of dissolution of the deposited metals [33]. Cr was least efficiently cleared from the lungs and 6 weeks after termination of exposure, 39% of the deposited Cr remained in the lungs [33]. In this study, we hypothesized that the translocation of dissolved metals from the lungs to the testes could directly affect spermatogenesis and decrease DSP. We therefore analyzed the metal content in the testes of Brown Norway rats. Metal content in the testes of exposed rats were not significantly elevated compared to the air controls. The metal contents were present at concentrations below the limit of detection (2.5 μg/kg for chromium) and the assessed metals were also found in the control rats, reflecting significant background levels, which may hamper the ability to detect treatment-related effects. In addition, we were only able to include 4–6 samples per group for the metal analyses, and only Brown Norway rats at the 12 week time point were assessed.

### Diet

Obesity and diets high in fat have been associated with chronic low-grade inflammation characterized by increased blood levels of PMNs and inflammatory cytokines, both factors which may affect sperm cells and their production [45, 46]. The results presented here gave no indications that the HF diet was disruptive to DSP. HF diet did, however, interact with GMA-SS exposure in relation to PNM influx for the Sprague Dawley rats, as discussed above. Moreover, serum testosterone levels were significantly lower in the HF diet Sprague Dawley rats. Increased adiposity is associated with lowering of testosterone levels because testosterone is can be converted to estradiol by aromatase expressed in adipose tissue [47]. Increased estrogen levels may subsequently inhibit the release of Gonadotropin-releasing hormone (GnRH) from the hypothalamus via negative feed-back, which may suppress Luteinizing hormone (LH) and Follicle stimulating hormone (FSH) production, ultimately reducing testosterone production [48]. The lower testosterone levels in the HF diet rats was not associated with decreased DSP in the present study. In rodents, normal spermatogenesis may proceed even after blockage of LH signaling, in the presence only the residual testosterone amounting to approximately 2% of control levels [49, 50]. The HF diet was not associated with changes in testosterone levels in the Brown Norway rats, even if this diet increased body weight gain significantly, indicating that this strain may be less sensitive to perturbation of hormonal levels by a diet high in fats, but it cannot be excluded that the high variability in testosterone levels might have masked hormonal changes. The GMA-SS exposure did not affect testosterone levels, but also here variation may have masked changes.

### Strain

Inbred strains of rodents are genetically more uniform compared to outbred strains, therefore outbred strains may display larger phenotypic variation, which might lower statistical power [27, 30]. Sperm quality and male reproductive capacity as well as sensitivity to chemical exposure may also differ with genotype and therefore between in- and outbred strains [28, 29]. Brown Norway rats often exhibit primary testicular defects characterized by decreased Sertoli cell function, lowered seminiferous tubule volume and low sperm content, and low serum testosterone levels [51], and also displayed somewhat lower testosterone levels compared to the Sprague Dawley rats in the present study. In fact, two homogenized testes samples from the Brown Norway rats were removed from the study because the samples were completely void of sperm cells. Such artefacts in sperm counts and testosterone background levels of Brown Norway rats may hamper our ability to detect slight changes to these endpoints. These characteristics of the Brown Norway strain has forwarded to a proposal of this strain as a model for reproductive ageing in man, specifically due to the occurrence of phenotypic decrements with age [51] as they may mimic the phenotypic variation seen in humans, e.g. in welders. Interestingly, the inbred Brown Norway rat has previously shown much more sensitivity to disruption of sperm production by irradiation and the chemotherapy drug procarbazine than the outbred Sprague Dawley rat [41].

In the present study, the two rat strains responded differentially to the welding exposure and the HF diet relative to the studied outcomes. In Brown Norway rats, lung inflammation lasted longer and PMN influx even seemed to increase during the recovery period. The HF diet was associated with increased weight gain in both strains, but only in the Sprague Dawley strain was weight gain overtly depressed by the GMA-SS exposure, in the group on regular diet. The effects on DSP and testosterone levels were also much more pronounced in the Sprague Dawley rats. In this strain DSP was depressed in both of the GMA-SS groups at 12 weeks compared to only the GMA-SS + HF group in the Brown Norway rats. At 24 weeks the Sprague Dawley strain furthermore presented with significant depression of testes weights following exposure to GMA-SS. Testosterone levels was depressed throughout the study period in the HF groups in the Sprague Dawley strain, but only at 24 weeks in the Brown Norway groups, and then only in the overall two-way ANOVA. Overall, the studied outcomes related to reproduction seemed much more prone to disruption in the Sprague Dawley compared to the Brown Norway strain, even if the latter mounted a much more protracted inflammatory response in the lungs. These findings provide a good example of differential toxicity between strains and furthermore demonstrates that Sprague Dawley strain may not be the least sensitive animal model for studies of male reproductive toxicity.

The observation that airway exposure to welding fumes decreased sperm production not only following intratracheal instillation [21] but also after inhalation exposure and in two strains of rats adds to existing evidence that welding fumes pose a hazard to the male reproductive system. For humans, it is reassuring that the observed effects may be transient, albeit the follow-up period was too short to evaluate whether the decreased testicular weights would recover. It should also be kept in mind that in the occupational setting welders often weld on a daily basis, and that the effect of long-term exposure was not assessed in the present study.

## Conclusion

In conclusion, inhalation of GMA-SS welding particles reduced DSP in two strains of rats, but overall, we did not find evidence to support that this was mediated by GMA-SS-induced lung inflammation. HF diet increased serum testosterone levels, without affecting DSP. There was some indication of interaction between GMA-SS exposure and intake of the HF diet in the Brown Norway strain, as sperm production was only reduced in the GMA-SS + HF diet group, however without corresponding interaction in other assessed parameters. Testicular Cr levels could also not be associated to changes in DSP. Therefore, no clear mechanism-of-action of the reduced DSP could be identified. The studied reproductive outcomes seemed much more prone to disruption in the Sprague Dawley compared to the Brown Norway strain, even if the latter mounted a more protracted inflammatory response in the lungs, indicative of differential reproductive toxicity between strains.

## Supplementary information

**Additional file 1: Figure** **S1.** Metal content of Cr, Ni, Mn, Fe and Cu in the testes of Brown Norway rats at week 12 following 5 weeks inhalation to 20 mg/m^3^ GMA-SS welding fumes. Mean ± standard deviation (*n* = 5–6).

**Additional file 2: Table S1.** Bronchoalveolar lavage fluid cell counts; *p* values of the two-way ANOVA. **Table S2.** Inflammatory cytokines; *p* values of the two-way ANOVA. **Table S3.** Testis parameters; *p* values of the two-way ANOVA. **Table S4.** Body weights and body weight gain; *p* values of the two-way ANOVA.

## Data Availability

Datasets used and/or analyzed during the current study are available from the corresponding author on reasonable request.

## Abbreviations

AM: Alveolar macrophages
BALF: Bronchoalveolar lavage fluid
DSP: Daily sperm production
FSH: Follicle stimulating hormone
GMA-MS: Gas metal arc-mild steel
GMA-SS: Gas metal arc-stainless steel
GnRH: Gonadotropin-releasing hormone
HF: High fat diet
HPG-axis: Hypothalamic-pituitary-gonadal axis
IARC: International Agency for Research on Cancer
LH: Luteinizing hormone
MMA-HS: Manual metal arc-hard surfacing
NIOSH: National Institute for Occupational Safety and Health
PMN: Neutrophils
Reg: Regular diet
SC/G_testes_: Sperm content per gram of testes
