# Supplementary figures and images for: Inhalation of welding fumes reduced sperm counts and high fat diet reduced testosterone levels; differential effects in Sprague Dawley and Brown Norway rats
Source: Part Fibre Toxicol. 2020 Jan 10;17:2. doi: 10.1186/s12989-019-0334-0 (PMC6954601; doi:10.1186/s12989-019-0334-0)

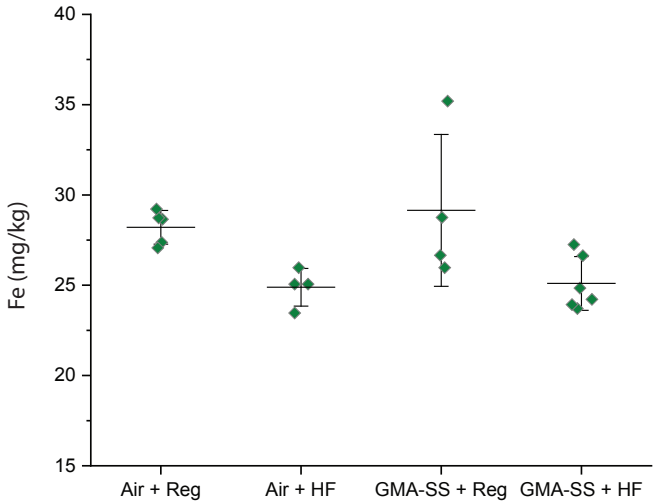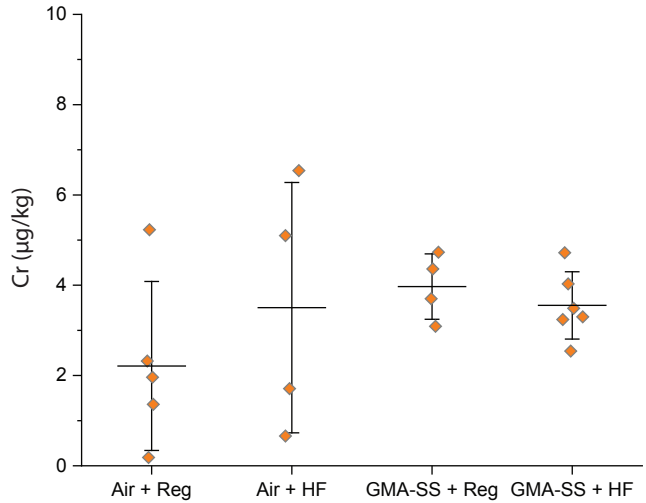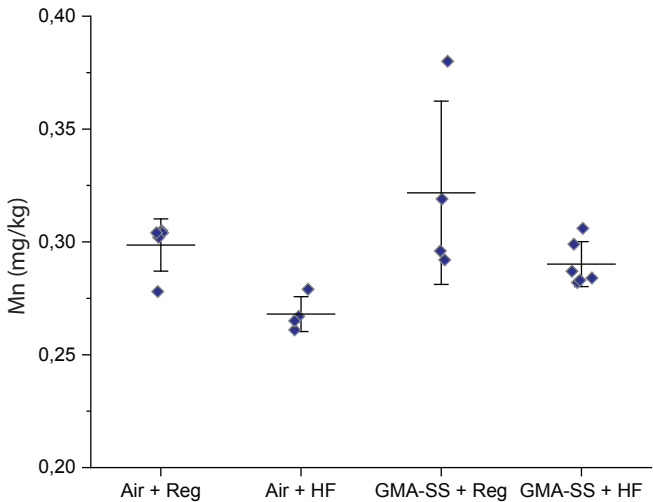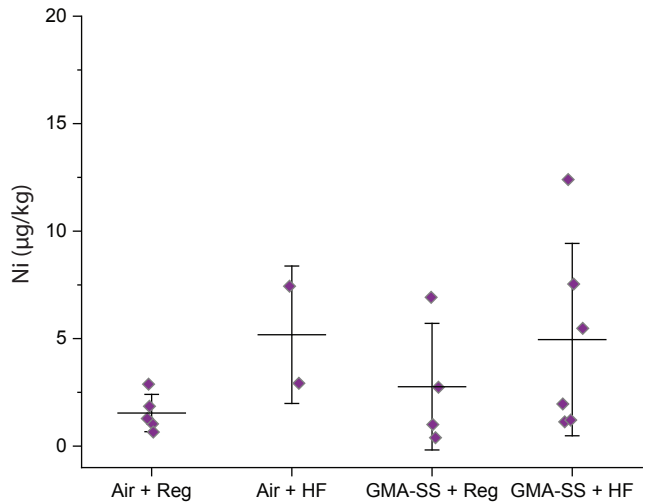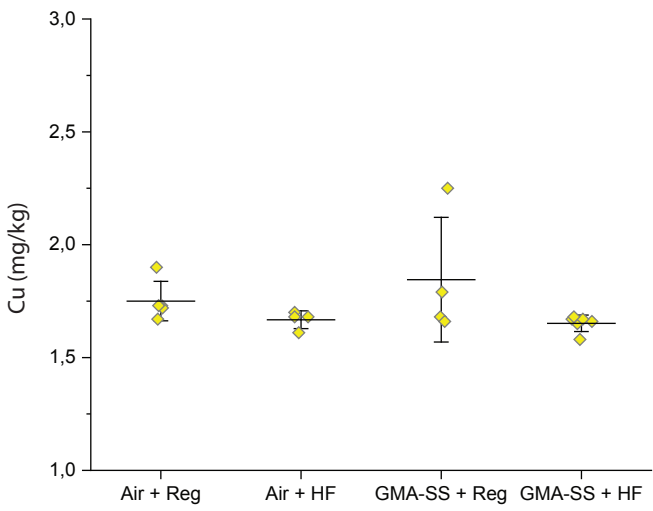

Supplement: Supplementary file 1 — Additional file 1: Figure S1. Metal content of Cr, Ni, Mn, Fe and Cu in the testes of Brown Norway rats at week 12 following 5 weeks inhalation to 20 mg/m3 GMA-SS welding fumes. Mean ± standard deviation (n = 5–6). [file 12989_2019_334_MOESM1_ESM.pdf]
