# Supplementary material for: Inhalation of welding fumes reduced sperm counts and high fat diet reduced testosterone levels; differential effects in Sprague Dawley and Brown Norway rats
Source: Part Fibre Toxicol. 2020 Jan 10;17:2. doi: 10.1186/s12989-019-0334-0 (PMC6954601; doi:10.1186/s12989-019-0334-0)
Supplement: Supplementary file 2 — Additional file 2: Table S1. Bronchoalveolar lavage fluid cell counts; p values of the two-way ANOVA. Table S2. Inflammatory cytokines; p values of the two-way ANOVA. Table S3. Testis parameters; p values of the two-way ANOVA. Table S4. Body weights and body weight gain; p values of the two-way ANOVA. [file 12989_2019_334_MOESM2_ESM.docx]

| **Table S1.** Bronchoalveolar lavage fluid cell counts; p values of the two-way ANOVA | | |
| --- | --- | --- |
| Response | Variable | P |
| Sprague Dawley: 12 weeks – exposure | | |
| BALF total cells | GMA-SS exposure  diet  GMA-SS x diet | < 0.001***  0.15  0.13 |
| BALF AM | GMA-SS exposure  diet  GMA-SS x diet | < 0.001***  0.43  0.39 |
| BALF PMN | GMA-SS exposure  diet  GMA-SS x diet | < 0.001***  < 0.001***  < 0.001*** |
| Sprague Dawley: 24 weeks – recovery | | |
| BALF total cells | GMA-SS exposure  diet  GMA-SS x diet | 0.02*  0.03*  0.79 |
| BALF AM | GMA-SS exposure  diet  GMA-SS x diet | 0.44  0.53  0.36 |
| BALF PMN | GMA-SS exposure  diet  GMA-SS x diet | 0.09  0.41  0.41 |
| Brown Norway: 12 weeks – exposure | | |
| BALF total cells | GMA-SS exposure  diet  GMA-SS x diet | < 0.001***  0.29  < 0.001*** |
| BALF AM | GMA-SS exposure  diet  GMA-SS x diet | < 0.001***  0.60  < 0.001*** |
| BALF PMN | GMA-SS exposure  diet  GMA-SS x diet | < 0.001***  0.03*  0.04* |
| Brown Norway 24 weeks – recovery | | |
| BALF total cells | GMA-SS exposure  diet  GMA-SS x diet | < 0.001***  0.93  0.40 |
| BALF AM | GMA-SS exposure  diet  GMA-SS x diet | < 0.001***  0.58  0.19 |
| BALF PMN | GMA-SS exposure  diet  GMA-SS x diet | < 0.001***  0.31  0.36 |

**Table S2.** Inflammatory cytokines; p values of the two-way ANOVA

| Response | Variable | P |  |
| --- | --- | --- | --- |
| Sprague Dawley: 12 weeks – exposure |  | | |
| IL-6 | GMA-SS exposure  diet  GMA-SS x diet | 0.11  0.02*  0.43 |  |
| CRP | GMA-SS exposure  diet  GMA-SS x diet | 0.80  1  0.45 |  |
| MCP-1 | GMA-SS exposure  diet  GMA-SS x diet | 0.29  0.19  0.09 |  |
| TNFa | GMA-SS exposure  diet  GMA-SS x diet | 0.84  0.92  0.51 |  |
| Sprague Dawley: 24 weeks – recovery |  | | |
| IL-6 | GMA-SS exposure  diet  GMA-SS x diet | 0.70  0.93  0.01* |  |
| CRP | GMA-SS exposure  diet  GMA-SS x diet | 0.17  0.08  0.02 * |  |
| MCP-1 | GMA-SS exposure  diet  GMA-SS x diet | 0.04  0.11  0.99 |  |
| TNFa | GMA-SS exposure  diet  GMA-SS x diet | 0.26  0.11  0.78 |  |

| **Table S3.** Testis parameters; p values of the two-way ANOVA | | |
| --- | --- | --- |
| Response | Variable | P |
| Sprague Dawley: 12 weeks – exposure | | |
| DSP | GMA-SS exposure  diet  GMA-SS x diet | < 0.001***  0.87  0.34 |
| SC/G_testes_ | GMA-SS exposure  diet  GMA-SS x diet | 0.003**  0.29  0.49 |
| Organ weight | GMA-SS exposure  diet  GMA-SS x diet | 0.37  0.09  0.9 |
| Testosterone | GMA-SS exposure  diet  GMA-SS x diet | 0.79  < 0.001***  0.10 |
| Sprague Dawley: 24 weeks – recovery | | |
| DSP | GMA-SS exposure  diet  GMA-SS x diet | 0.06  0.37  0.82 |
| SC/G_testes_ | GMA-SS exposure  diet  GMA-SS x diet | 0.81  0.71  0.64 |
| Organ weight | GMA-SS exposure  diet  GMA-SS x diet | 0.006**  0.26  0.26 |
| Testosterone | GMA-SS exposure  diet  GMA-SS x diet | 0.08  < 0.001***  0.37 |
| Brown Norway: 12 weeks – exposure | | |
| DSP | GMA-SS exposure  diet  GMA-SS x diet | 0.04*  0.05  0.08 |
| SC/G_testes_ | GMA-SS exposure  diet  GMA-SS x diet | 0.04*  0.92  0.23 |
| Organ weight | GMA-SS exposure  diet  GMA-SS x diet | 0.77  < 0.001**  0.19 |
| Testosterone | GMA-SS exposure  diet  GMA-SS x diet | 0.36  0.51  0.83 |
| Brown Norway 24 weeks – recovery | | |
| DSP | GMA-SS exposure  diet  GMA-SS x diet | 0.46  0.86  0.19 |
| SC/G_testes_ | GMA-SS exposure  diet  GMA-SS x diet | 0.35  0.49  0.29 |
| Organ weight | GMA-SS exposure  diet  GMA-SS x diet | 0.35  0.35  0.35 |
| Testosterone | GMA-SS exposure  diet  GMA-SS x diet | 0.71  0.02*  0.66 |

| **Table S4.** Body weights and body weight gain; p values of the two-way ANOVA | | |
| --- | --- | --- |
| Response | Variable | P |
| Sprague Dawley: 12 weeks – exposure | | |
| Body weights | GMA-SS exposure  diet  GMA-SS x diet | < 0.001***  < 0.001***  0.07 |
| Sprague Dawley: 24 weeks – recovery | | |
| Body weights | GMA-SS exposure  diet  GMA-SS x diet | 0.03  < 0.001***  0.001*** |
| Brown Norway: 12 weeks – exposure | | |
| Body weights | GMA-SS exposure  diet  GMA-SS x diet | 0.69  0.09  0.92 |
| Brown Norway 24 weeks – recovery | | |
| Body weights | GMA-SS exposure  diet  GMA-SS x diet | 0.19  < 0.001***  0.49 |
| Sprague Dawley: 12 weeks – exposure | | |
| Body weight gain | GMA-SS exposure  diet  GMA-SS x diet | 0.001***  0.000***  0.038 |
| Sprague Dawley: 24 weeks – recovery | | |
| Body weight gain | GMA-SS exposure  diet  GMA-SS x diet | 0.027*  0.000***  0.002*** |
| Brown Norway: 12 weeks – exposure | | |
| Body weight gain | GMA-SS exposure  diet  GMA-SS x diet | 0.42  0.000***  0.57 |
| Brown Norway 24 weeks – recovery | | |
| Body weight gain | GMA-SS exposure  diet  GMA-SS x diet | 0.32  0.000***  0.79 |
